# Association between psychological stress, anxiety and oral health status among college students during the Omicron wave: a cross-sectional study

**DOI:** 10.1186/s12903-023-03151-3

**Published:** 2023-07-10

**Authors:** Rongkai Cao, Junyu Lai, Xiaoxin Fu, Piaopiao Qiu, Jinghong Chen, Weicai Liu

**Affiliations:** 1 Stomatological Hospital and Dental School of Tongji University, Shanghai Engineering Research Center of Tooth Restoration and Regeneration, Shanghai, China; 2 Shanghai Mental Health Center, School of Medicine, Shanghai Jiao Tong University, Shanghai, China; 3 Faculty of Education, East China Normal University, Shanghai, China

**Keywords:** COVID-19, Psychological stress, Anxiety, Oral health status, Association

## Abstract

**Background:**

Within 3 years of the COVID-19 pandemic, increasing interest has been given to its potential influence on health status due to lockdowns caused by the pandemic. However, the impact is inadequately understood, especially for college students. This study aimed to investigate the potential association between psychological stress, anxiety and oral health of college students during the Omicron wave of the COVID-19 pandemic.

**Methods:**

An online survey with measurements of psychological stress, anxiety and oral health was completed by 1770 Chinese college students. The Perceived Stress Scale-14 (PSS-14) and Generalized Anxiety Disorder-7 (GAD-7) were used to measure psychological stress and anxiety, respectively. Oral health status was self-reported including toothache, gingival bleeding, and oral ulcer. Multivariable logistic regressions were performed to determine underlying associations for outcome variables. Structural equation modeling (SEM) was performed to confirm the relationship between mental and oral health status.

**Results:**

Of the 1770 subjects, 39.2% presented high psychological stress and only 41.2% expressed no anxiety. A significant association was found between psychological stress, anxiety and oral health status. Anxiety has significant impacts on toothache (OR = 0.36; 95%CI: 0.23–0.55; *p* < 0.01), gingival bleeding (OR = 0.43; 95%CI: 0.29–0.65; *p* < 0.01), and oral ulcer (OR = 0.54; 95%CI: 0.36–0.80; *p* < 0.01). Anxiety significantly mediated the association between psychological stress and self-reported oral symptoms.

**Conclusions:**

Anxiety may be a significant risk indicator for mental health among college students and demonstrates a significant relationship with the occurrence of self-reported oral symptoms. Concerns about academic and life changes caused by the pandemic were the two most significant sources of stress.

## Introduction

It has been three years since COVID-19, the biggest public health crisis in a century, was designated as a global pandemic by the World Health Organization (WHO) on March 11, 2020 [1]. Due to the strict and effective public health measures taken by the government and the support provided by the international community, the spread of COVID-19 in China was effectively controlled [2, 3]. However, as the Omicron variant emerged and spread globally at an alarming speed, greater public health concerns have been raised around the world [4]. With the second wave of epidemic which originated in March 2022 coming to an end, many problems worth considering emerged.

One of the most critical issues is the unprecedented and disruptive changes that have taken place in the daily life of college students. Each country has taken corresponding measures to deal with the COVID-19 infection based on its situation. In China, measures to reduce the movement of people include canceling mass gathering activities, postponing the spring semester and controlling transportation capacity [5]. Results from previous studies indicated that college students were especially vulnerable to those adverse outcomes during the pandemic [6]. Greater anxiety among college students may be due to concerns about grades during online learning, inadequate communication with teachers and difficulty in concentrating. Subjects influenced by the COVID-19 pandemic may present a high epidemiological burden of stress, depression, sleep disorders, anxiety disorders, emotional disturbance, and more mental health problems [7]. Accordingly, mental health problems are increasing in number and severity on university campuses. The increased psychological stress and anxiety in college students is becoming a serious problem and brought great concerns across the globe. An interview survey conducted in the United States showed that 71% of college students reported increased psychological stress and anxiety because of the COVID-19 outbreak [8]. A meta-analysis conducted during the COVID-19 pandemic showed the occurrence of anxiety reached 13.9% and significantly higher than the rate before the outbreak (5%) [9]. Another large-scale survey recruiting 821,218 college students in China concluded that the prevalence of stress, anxiety, and depressive symptoms were 34.9%, 11.0%, and 21.0%, respectively [10]. Reasons for the increased psychological stress and anxiety among students in university during COVID-19 include increased concerns about academic performance, fear and worry about their health status, decreased social interactions due to physical distancing, and greater access to information through social media.

Psychological stress can result in serious health problems including cardiovascular disease and gastrointestinal problems [11]. When faced with harmful stimuli (known as stressors), it includes the response and adjustment made by the human body to maintain homeostasis, as well as the discussion of relative health and disease problems. Anxiety is one of the most common emotional symptoms of stress. In addition, a previous study has demonstrated that high anxiety was statistically associated with lower self-reported course grades and persistence in the Biology major [12]. While psychological stress and anxiety may differ, it is clear that both are major challenges facing college students. The increased mental health problems may harm the quality of life and health among college students [13]. Moreover, the relationship between mental health and oral health has been demonstrated in the literature [14–16]. For example, psychological adversities such as depression and generalized anxiety may harm oral health [14]. Results of another previous study showed an association between stress and periodontitis, indicating the necessity of attention to psychological stress in the management of oral health conditions [15]. Moreover, researchers also proposed that improving oral health will contribute to improving mental health outcomes [16].

Oral health is characterized by a comfortable functional dentition with an appearance that allows the improved performance of social function and daily activities, without physical, psychological, or social disturbances [17]. Oral health-related quality of life, influenced by oral hygiene status, was significantly associated with emotional distress [18]. Results of a previous meta-analysis indicated that mental health was associated with increased dental caries as well as greater tooth loss [19]. Depression has also been demonstrated to adversely influence periodontal health status by causing neglect of tooth brushing [20]. In addition, generalized anxiety may be associated with oral ulcers and influence the quality of tooth brushing [21].

SEM is an approach to build, estimate and test causal relationship models [22]. Compared with traditional statistical methods, the integration of multiple regression, factor analysis, path analysis, and covariance analysis allows SEM to model the relationship among multiple factors, which clearly shows the relationship between one single indicator and the result variables as well as among single indicators, with allowing to ignore the measurement error [23]. This statistical analysis is more conducive to understanding our more complex real world and discovering more hidden relationships. In addition, since mental health level is difficult to be directly measured, anxiety and stress level was chosen as the main indirect indicators to observe the mental status, and other possible related factors as secondary indicators.

The health condition of college students may be more vulnerable during online learning periods due to the lockdowns [24]. Accordingly, it is of particular urgency and importance to focus on both mental and oral health status in this population. There are limited research investigating the adverse influence of the COVID-19 pandemic on psychological stress, anxiety and oral health status among college students in China, especially for cross-sectional studies with large sample size. In addition, the relationship between them during the pandemic and the external factors that cause oral problems by triggering anxiety and stress remain to be elucidated. Based on the findings above, this study aimed to conduct an online cross-sectional study to investigate the potential association between psychological stress, anxiety and oral health status among college students during the Omicron wave of the COVID-19 pandemic.

## Methods

### Study design

An online questionnaire was completed on the online platform, targeting current college students, ranging from September 23rd to October 23rd in 2022.

### Participants

A cross-sectional study was conducted to recruit current college students who were residents of China at the time when they completed the survey. Inclusion criteria encompassed being at least 18 years of age and a current student at the university. Ethical approval for the present study was obtained from the Ethics Committee of the School & Hospital of Stomatology, Tongji University (approval number [2022]-SR-19). Subjects who participated in the study were informed about the objectives, inclusion criteria, anonymity of data collection and the right to withdraw from study participation. Subjects could only complete the questionnaire if they consented to participate in the survey.

### Measurements

#### Demographic characteristics

Questions in the demographics section collected information on the participants’ age, major, gender, education level, parental education level, and whether they are fresh graduates. Major was divided into liberal arts and science according to previous research [25]. The education level of college students included undergraduate, master and doctorate degrees. To further reflect the gap in family educational background, high school was used as the cutoff for parental education level.

#### Perceived stress scale 14

Psychological stress was evaluated using the 14-item version of the Perceived Stress Scale [26]. PSS-14 evaluates the psychological stress level experienced during the last month under certain life circumstances. Each item was assessed by a five-point Likert scale ranging from 0 (“never”) to 4 (“very often”). The scores range from 0 to 56. Based on PSS-14 scores, psychological stress was divided into three levels. 0 to 28 indicates normal pressure level, 29 to 42 indicates high pressure level, and scores more than 42 are regarded as super pressure. The reliability coefficient of PSS-14 demonstrated acceptable internal consistency [27]. The Chinese version of PSS-14 has also been validated in the literature [28]. In addition, the potential sources of psychological stress were also investigated.

#### Generalized anxiety disorder 7

The anxiety of college students was measured by GAD-7, which is a screening scale containing 7 questions for generalized anxiety disorder that measures the anxiety symptoms of individuals during the past 2 weeks [29]. Each question had four options, namely—not at all, several days, more than half the days, nearly every day—with a four-point Likert scale of 0, 1, 2, 3, respectively. The gradation of GAD-7 based upon the scores was divided into four levels: 0 to 4 = no anxiety; 5 to 9 = mild anxiety; 10 to 14 = moderate anxiety; 15 to 21 = severe anxiety. The reliability and validity of the Chinese version of GAD-7 have also been proven in the previous study [30].

#### Oral health status

In this study, oral health status was considered as the dependent variable. Self-reported symptoms of toothache, gingival bleeding and oral ulcer were selected by the investigators as three main parameters that intended to measure the oral health status of college students in the present study. College Students were asked to report if they have toothache, gingival bleeding and oral ulcer symptoms (0 = no; 1 = yes) during the Omicron wave of the COVID-19 pandemic.

### Variables

#### Anxiety

0 to 28 indicates normal pressure level, 29 to 42 indicates high pressure level and scores more than 42 are regarded as super pressure.

#### Stress

Based upon the scores of GAD-7: 0 to 4 = no anxiety; 5 to 9 = mild anxiety; 10 to 14 = moderate anxiety; 15 to 21 = severe anxiety.

#### Oral health status

College Students were asked to report if they have toothache, gingival bleeding and oral ulcer symptoms (0 = no; 1 = yes).

#### Others

Demographic characteristics such as age, major, gender, education level, parental education level and whether they are fresh graduates were collected. In addition, the sources of stress were also taken into account.

### Bias

The questionnaire was posted online and was accessible to all university students. In addition, SEM was used to analyze the data, which can ignore the influence of data bias.

### Study size

Due to the instability of the epidemic, we did not directly calculate the sample size. However, the sample size of this study is sufficient, not only because it exceeds the sample size of other similar studies related to mental health during the epidemic [31–33], but also because it significantly proved the significant association among relevant indicators based on the result.

### Statistical methods

The statistical analysis was performed with SPSS software 27.0 (IBM Corp., Armonk, NY, USA). Data about the qualitative characteristics were expressed as percentage values and measurement data were described by the average and standard deviation. Differences in self-reported symptoms of toothache, gingival bleeding, and oral ulcer were compared between participants using Chi-squared tests for all variables except age (*t*-test). Multivariable logistic regression analysis was used to investigate the underlying relationship between psychological stress, anxiety, and oral health status. Logistic regression models were adjusted for sex, age, major, education level, parental education level, and fresh graduates. All variables were considered categorical variables except age, which was regarded as a continuous variable. Results from the logistic regression analyses are presented as OR with 95% CI. The *p*-value < 0.05 was considered at a significant level. In addition to the multivariate logistic regression, we also conducted SEM in Mplus 8.3 to disentangle the association between stress, anxiety, and oral health status. SEM combines factor analysis and path analysis, which not only can include observed and latent variables but also reveal the direct and indirect effects [34]. Self-reported oral symptoms are a latent variable that consists of three observed variables: toothache, gingival bleeding and oral ulcer. Self-reported oral symptoms can not be measured directly but are estimated from measured variables (toothache, gingival bleeding and oral ulcer) in the model. The goodness-of-fit model of SEM was assessed using the Root Mean Squared Error of Approximation (RMSEA), Tucker-Lewis Index (TLI), Comparative Fit Index (CFI), and Standardized Root Mean Square Residual (SRMR). The model fit was considered acceptable when RMSEA < 0.1, CFI and TLI > 0.90, and SRMR < 0.08 [35].

## Results

Ranging from September 23rd to October 23rd in 2022, 1,812 subjects were collected, of which 1,770 (97.68%) met the criteria for data integrity and inclusion, including 644 (63.6%) male subjects and 1126 (63.6%) female subjects. Age ranged from 18 to 30, with an average of 22.6 years. The sample included both undergraduate (*n* = 983, 55.5%) and graduate students (*n* = 787, 44.5%). Most of the subjects’ parents (59.1%) had a high school degree or below. 505 (28.5%) of the subjects were fresh graduates and the number of students who majored in liberal arts and science was 730 (41.2%) and 1040 (58.8%), respectively (Table 1).

**Table 1:** Sample characteristics

Characteristics	Category	Total	Toothache			Gingival bleeding			Oral ulcer		
			**Yes**	**No**	***p***	**Yes**	**No**	***p***	**Yes**	**No**	***p***
Age	Mean (SD)	22.6(2.5)	22.4 (2.5)	22.7 (2.5)	0.03*	22.4 (2.4)	22.7 (2.6)	< 0.01**	22.5 (2.6)	22.7 (2.5)	0.30
Gender	Male	644(36.4%)	148 (34.2%)	496 (37.1%)	0.27	200 (35.4%)	444 (36.8%)	0.56	257 (39.2%)	387 (34.7%)	0.06
	Female	1126(63.6%)	285 (65.8%)	841 (62.9%)		365 (64.6%)	761 (63.2%)		399 (60.8%)	727 (65.3%)	
Major	Liberal arts	730(41.2%)	200 (46.2%)	530 (39.6%)	0.02*	254 (45.0%)	476 (39.5%)	0.03*	268 (40.9%)	462 (41.5%)	0.80
	Science	1040(58.8%)	233 (53.8%)	807 (60.4%)		311 (55.0%)	729 (60.5%)		388 (59.1%)	652 (58.5%)	
Education	Undergraduate	983(55.5%)	244 (56.4%)	739 (55.3%)	0.62	330 (58.4%)	653 (54.2%)	0.22	364 (55.5%)	619 (55.6%)	0.71
	Master	658(37.2%)	154 (35.6%)	504 (37.7%)		194 (34.3%)	464 (38.5%)		240 (36.6%)	418 (37.5%)	
	Doctor	129(7.3%)	35 (8.1%)	94 (7.0%)		41 (7.3%)	88 (7.3%)		52 (7.9%)	77 (6.9%)	
Parental education level	> High school	724(40.9%)	164 (37.9%)	560 (41.9%)	0.14	223 (39.5%)	501 (41.6%)	0.40	256 (39.0%)	468 (42.0%)	0.22
	≤ High school	1046(59.1%)	269 (62.1%)	777 (58.1%)		342 (60.5%)	704 (58.4%)		400 (61.0%)	646 (58.0%)	
Fresh graduates	Yes	505(28.5%)	112 (25.9%)	393 (29.4%)	0.16	153 (27.1%)	352 (29.2%)	0.35	171 (26.1%)	334 (30.0%)	0.08
	No	1265(71.5%)	321 (74.1%)	944 (70.6%)		412 (72.9%)	853 (70.8%)		485 (73.9%)	780 (70.0%)	
Stress	Normal	1077(60.8%)	228 (52.7%)	849 (63.5%)	< 0.01**	316 (55.9%)	761 (63.2%)	< 0.01**	375 (57.2%)	702 (63.0%)	< 0.01**
	High	646(36.5%)	189 (43.6%)	457 (34.2%)		227 (40.2%)	419 (34.8%)		256 (39.0%)	390 (35.0%)	
	Super	47(2.7%)	16 (3.7%)	31 (2.3%)		22 (3.9%)	25 (2.1%)		25 (3.8%)	22 (2.0%)	
Anxiety	No	730(41.2%)	121 (27.9%)	609 (45.5%)	< 0.01**	187 (33.1%)	543 (45.1%)	< 0.01**	227 (34.6%)	503 (45.2%)	< 0.01**
	Mild	566(32.0%)	150 (34.6%)	416 (31.3%)		181 (32.0%)	385 (32.0%)		211 (32.2%)	355 (31.9%)	
	Moderate	308(17.4%)	101 (23.3%)	207 (15.5%)		121 (21.4%)	187 (15.5%)		139 (21.2%)	169 (15.2%)	
	Severe	166(9.4%)	61 (14.1%)	105 (7.9%)		76 (13.5%)	90 (7.5%)		79 (12.0%)	87 (7.8%)	

Chi-squared tests for gender, major, education, parental education level, fresh graduates, stress and anxiety; independent-sample t test for age

^*^p < 0.05; **p < 0.01

### Prevalence of psychological stress and anxiety

**Fig. 1:**
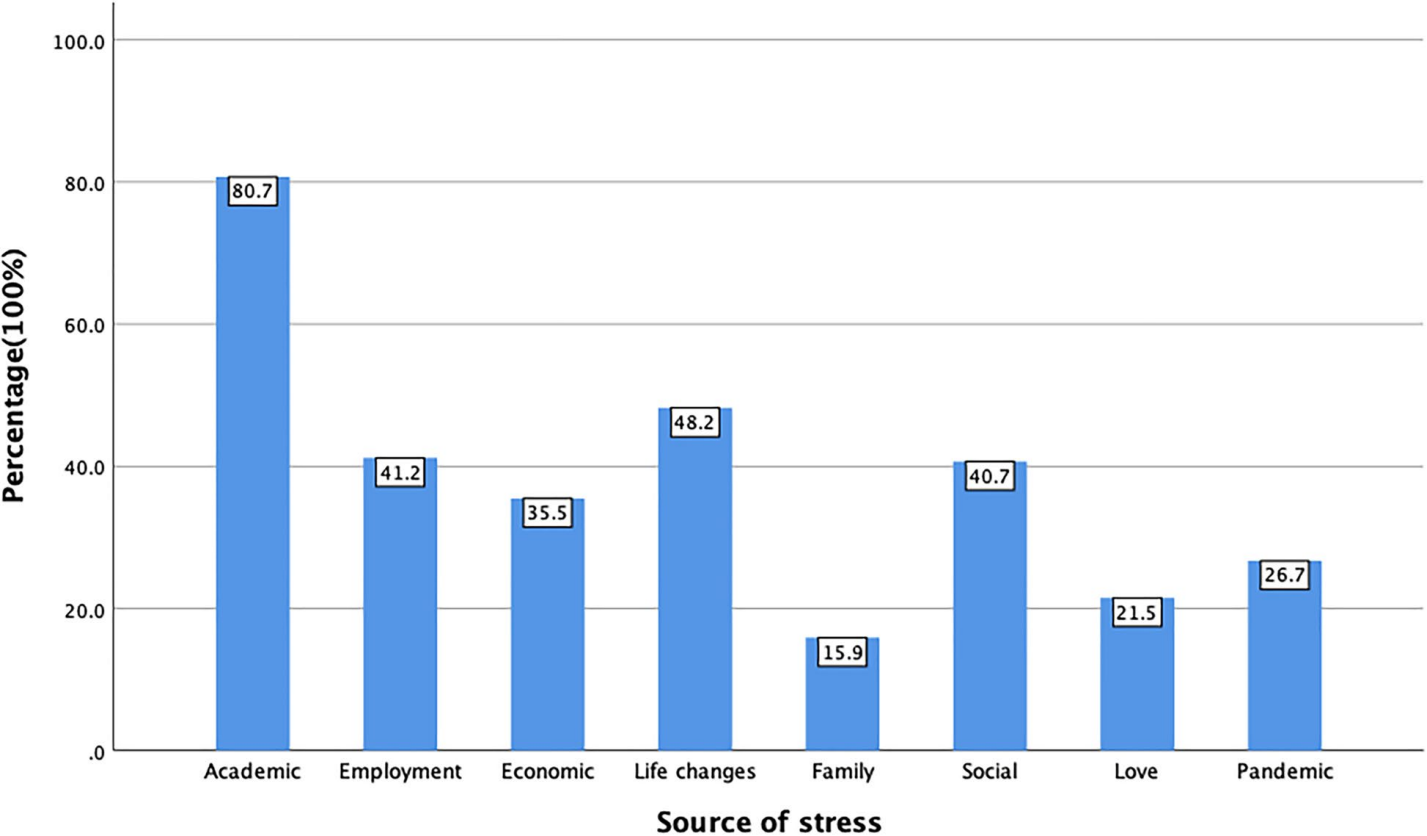
Source of psychological stress

Among the 1770 subjects included, normal psychological stress accounted for 60.8%, high psychological stress accounted for 36.5% and super psychological stress accounted for 2.7%. Academic stress was considered the most important factor which puzzled the mental health of college students (80.7%), other major sources of stress including employment stress (41.2%), economic stress (35.5%), living environment change stress (48.2%), and social stress (40.7%) (Fig. 1). Regarding anxiety, only 41.2% of students were classified as having no anxiety. The detection rates of mild, moderate, and severe anxiety were 32.0%, 17.4%, and 9.4%, respectively (Table 1).

### Oral health status

4.5% of the subjects reported symptoms of toothache. Students with oral ulcers and self-reported gingival bleeding were 37.1% and 31.9%, respectively. Students who were younger and majored in liberal arts are more likely to have toothache (*p* < 0.05) and gingival bleeding (*p* < 0.05). The results of Chi-squared tests indicated that anxiety was considered a significant influencing factor for the three main oral health parameters (*p* < 0.05). In addition, students with more psychological stress were also more likely to report toothache, gingival bleeding, and oral ulcer (*p* < 0.05) (Table 1).

### Association between psychological stress, anxiety and oral health status

After controlling several variables (that is, gender, age, major, parental education level, education level, and fresh graduates), the multivariate logistic regression analysis demonstrated a negative relationship between toothache, gingival bleeding, oral ulcer, and anxiety. Compared to students with severe anxiety, those without anxiety were less likely to have toothache (OR = 0.358, *p* < 0.01, gingival bleeding (OR = 0.431, *p* < 0.01), and oral ulcer (OR = 0.535, *p* < 0.01). However, stress was not found to significantly affect toothache, gingival bleeding, and oral ulcer in the logistic regression (Table 2).

**Table 2:** Multivariate logistic regression of factors associated with oral health status

Characteristics	Category	Dependent Variable: Oral Health Status								
		**Toothache**			**Gingival bleeding**			**Oral ulcer**		
		**WaldΧ**^**2**^	*p*	**OR (95%CI)**	**WaldΧ**^**2**^	***p***	**OR (95%CI)**	**WaldΧ**^**2**^	***p***	**OR (95%CI)**
Age	N/A	3.67	0.06	0.94 (0.088–1.00)	2.75	0.10	0.95 (0.89–1.01)	1.24	0.27	0.97 (0.91–1.03)
Gender	Male	0.11	0.74	0.96 (0.75–1.22)	0.03	0.87	1.018 (0.82–1.27)	3.88	< 0.05*	1.24 (1.00–1.53)
	Female									
Major	Science	3.37	0.07	1.25 (0.99–1.60)	2.27	0.13	1.19 (0.95–1.48)	< 0.01	0.97	1.00 (0.81–1.25)
	Liberal arts									
Education	Undergraduate	3.54	0.06	0.59 (0.34–1.02)	0.74	0.39	0.80 (0.48–1.33)	0.98	0.32	0.78 (0.48–1.27)
	Master	2.67	0.10	0.07 (0.43–1.08)	1.27	0.26	0.78 (0.51–1.20)	0.96	0.33	0.82 (0.54–1.23)
	Doctor									
Parental education level	≤ High school	2.06	0.15	0.85 (0.67–1.06)	0.50	0.48	0.93 (0.75–1.14)	2.03	0.15	0.86 (0.71–1.06)
	> High school									
Fresh graduates	Yes	0.43	0.51	0.91 (0.70–1.20)	0.12	0.73	0.96 (0.75–1.22)	1.18	0.28	0.88 (0.70–1.11)
	No									
Stress	Normal	0.03	0.86	1.06 (0.53–2.16)	0.14	0.70	0.88 (0.45–1.72)	0.71	0.40	0.75 (0.39–1.46)
	High	0.24	0.63	1.19 (0.60–2.35)	0.11	0.75	0.90 (0.47–1.72)	0.81	0.37	0.74 (0.38–1.42)
	Super									
Anxiety	No	21.87	< 0.01**	0.36 (0.23–0.55)	16.65	< 0.01**	0.43 (0.29–0.65)	9.43	< 0.01**	0.54 (0.36–0.80)
	Mild	4.79	0.03*	0.63 (0.42–0.95)	7.12	< 0.01**	0.58 (0.39–0.87)	2.92	0.09	0.71 (0.48–1.05)
	Moderate	0.60	0.44	0.85 (0.55–1.29)	1.01	0.31	0.81 (0.54–1.22)	< 0.01	0.95	0.98 (0.66–1.48)
	Severe									

The following covariates were controlled for age, gender, major, education, parental education level, fresh graduates

Reference group: Female, Liberal arts, Doctor, > High school, No fresh graduates, Super stress, Severe anxiety

^*^p < 0.05; **p < 0.01

### Structural equation model and its verification results

**Fig. 2:**
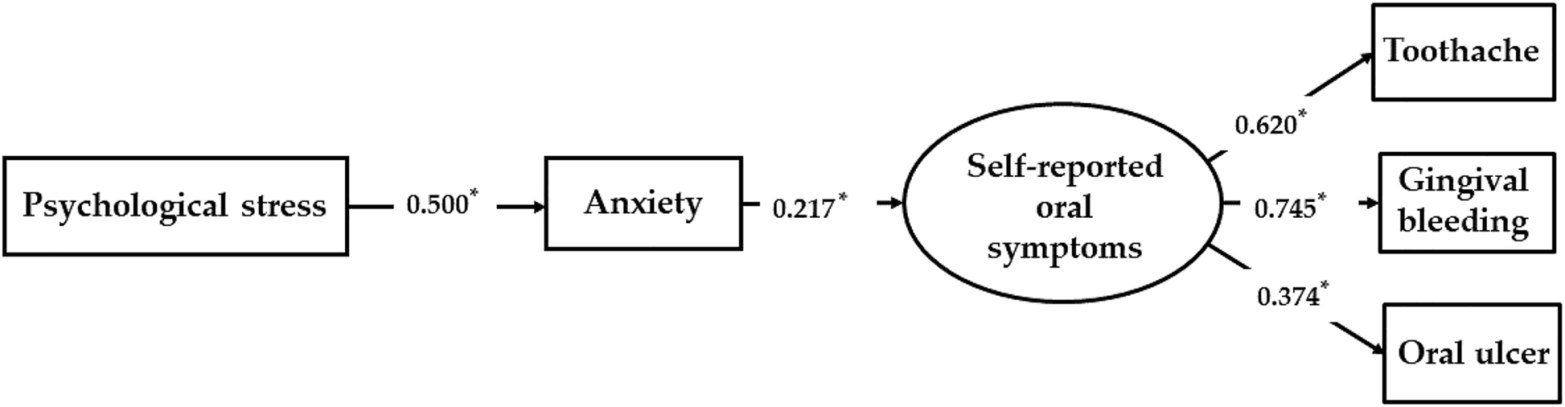
Standardized coefficients for the structural model testing the mediation effect of anxiety on the relationship between psychological stress and self-reported oral symptoms. *p < 0.05

After several rounds of model modification, a model was finally fitted from complex clinical data and most matched with the data. The main load coefficients of the model are almost significant (*p* < 0.01). The above results show that the model has a good fit with the data (CFI = 0.998, TLI = 0.995, RMSEA = 0.021, SRMR = 0.012). Figure 2 shows standardized coefficients for the structural model. Psychological stress had a significant effect on anxiety and anxiety significantly affected self-reported oral symptoms. Anxiety significantly mediated the relationship between psychological stress and self-reported oral symptoms.

## Discussion

The current study findings provide the prevalence of psychological stress, anxiety, and oral health among college students in China during the Omicron wave of the COVID-19 pandemic and investigate the underlying relationship between them. The results demonstrated a significant association between psychological stress, anxiety and the occurrence of self-reported oral symptoms among Chinese college students. Detection rates of psychological stress and anxiety among college students were 39.2% and 58.8%, respectively, which were higher than a previous study conducted in 2015 before the COVID-19 pandemic [36]. The results may account for the unprecedented severe changes caused by Omicron. To our knowledge, this is the first investigations to draw attention to the relationship between oral and mental health among Chinese college students during the Omicron wave of the COVID-19 pandemic.

Psychological stress is an unavoidable part of our life, which affects the physical health status of a person. Due to limited free time, long teaching hours, intense exams, and high workloads, college students are being placed in a greater level of stressful situations than the average representative of society [37–39]. Results of PSS-14 demonstrated the high perceived stress levels among college students. When assessing the source of stress, academic stress (80.7%) was considered the most significant factor, which is consistent with previous research conducted in 2015 [40]. During the lockdown, students are still required to study through the online platform and none of their academic research can be conducted normally, so students are under additional academic pressure. Accordingly, strategies designed to help college students relieve psychological stress during the Omicron pandemic should target the stress from academic sources of them.

The results of the GAD-7 showed that 41.2% of Chinese college students did not have anxiety. However, 32.0% of the subjects have mild anxiety levels, and 17.4% suffer from moderate anxiety. In addition, 9.4% of college students have suffered from severe anxiety during the Omicron pandemic. A previous study of Chinese college students conducted at the beginning of the COVID-19 pandemic indicated that 75.1% of students reported no anxiety, while the subjects with mild, moderate, and severe anxiety were 21.3%, 2.7%, and 0.9%, respectively [41]. The Omicron pandemic seems to have increased anxiety symptoms among college students during the last several months. The unprecedented threats provided by the spread of Omicron may account for this situation, since Omicron may combine with a higher community transmission and hospitalization load, which potentially overwhelm healthcare systems [42].

In the present study, the high prevalence of self-reported gingival bleeding and oral ulcer alerted that the oral health status of college students should be considered. Students with more anxiety are more likely to report subjective oral symptoms, which demonstrated an underlying association between anxiety and oral health status in the present study. All of the associations between anxiety and oral health status were statistically significant and possible explanations could be made for these associations. Those with less anxiety might have better lifestyle-related behaviors and oral health behaviors, which were associated with better oral health perception [43, 44]. There is some evidence that links anxiety with oral diseases. A previous investigation observed a statistically significant association between anxiety and gingival bleeding [45]. Folayan et al. conducted a cross-sectional study in Nigeria during the COVID-19 pandemic and indicated the odds of having an oral ulcer were higher as the generalized anxiety symptoms increased [46]. Furthermore, a previous study conducted in 2006 demonstrated that anxiety was significantly and independently related to perceived toothache [47]. Accordingly, toothache, gingival bleeding, and oral ulcer were selected as the main parameters for oral health status in the present study. Our results were consistent with previous investigations and proved the associations between self-reported symptoms and mental health. Therefore, to improve the self-assessment of health status, strategies should focus on the alleviation of anxiety among college students.

Although there was a significant correlation between stress and oral health, our study did not find that stress could significantly affect oral health in the logistic regression. However, through further analysis, we found that stress can further influence oral health by affecting anxiety using SEM. Stress is one of the significant predictors of anxiety [48]. Students who are anxious experience more stress and respond differently to stressors compared to those who are not anxious. Thus, a possible explanation is although students are under stress, individuals have different social support and respond differently to stressors [49, 50]. Some may not be in anxiety and thus their oral status will not be affected, while other students who are under high stress may trigger their anxiety [51]. Therefore, stress can further influence oral health by affecting anxiety. This result enlightens us to pay attention to the sources of stress in students and further promotes oral health by relieving their stress.

The results of this study found that the magnitude of psychological stress and anxiety among college students in China during the Omicron wave of the COVID-19 pandemic was concerning. An association was found between psychological stress, anxiety, and self-reported oral health status. Therefore, the increased focus on mental health should encompass oral health status. Currently, several studies have investigated the association between anxiety and oral health [19, 47]. However, there is limited information about how the relationship is mediated. These relationships are thought to be influenced by changes in immunity triggered by emotional responses, including cellular proliferation, or health-related risk behaviors caused by stressful situations, including a reduction in brushing teeth, or even both [45]. However, most of the research has focused on proximate causes like bacteria and immune mechanisms about the psychological factors and how they influence the host's immune response to bacteria. Further investigations are required to find out the biological mechanisms between mental and oral health and to help health promotion initiatives in designing effective programs.

The present study has some limitations. First of all, the use of an online survey may lead to some biased results and relatively low detection rates of psychological stress and anxiety, because the students who voluntarily filled in the survey may generally have good mental health status, and the actual oral conditions can not be assessed through an online survey. In addition, results in this study may not be applied to all college students in China since samples included several developed cities where students' mental health may be different from other regions. Another issue needed to be considered is that the survey was conducted at a relatively late stage of lockdown, and students' psychological stress and anxiety responses were in remission. Finally, oral health behavior such as the frequency of tooth brushing, use of fluoride and interdental cleaning device was not considered in this study. Other disease conditions, such as cardiovascular diseases and diabetes, were unknown, and causality was undetermined. Therefore, the survey only reflects the effect of the Omicron wave of the COVID-19 pandemic on oral and mental health status to a certain extent.

## Conclusions

Overall, the present study showed the magnitude of psychological stress and anxiety among college students in China is alarmingly high during the Omicron wave of the COVID-19 pandemic. A significant relationship was found between psychological stress, anxiety and the occurrence of self-reported oral health symptoms among college students. Before recommendations for policy and practice can be made, future longitudinal studies and randomized controlled trials need to be carried out to analyze the direction of the associations, and to identify mediating factors involved in these relationships.

## Data Availability

The data presented in this study are available on request from the corresponding author.

## Abbreviation

COVID-19: Corona Virus Disease 2019
